# The Impact of the Patras Carnival on the Course of the COVID-19 Pandemic and the Outbreak of Influenza A: A Multicenter Cross-Sectional Study

**DOI:** 10.3390/vaccines10091484

**Published:** 2022-09-06

**Authors:** Christos Sotiropoulos, Vasileios Loulelis, Anna Mastorakou, Eleni Adamopoulou, Theoharis Kortesas, Aimilia Dimitropoulou, Eleni Blika, Ioanna Papadimitriou-Olivgeri, Ilona Binenbaum

**Affiliations:** 1 Sotiropoulos Private Laboratory, 26222 Patras, Greece; 2 Loulelis Private Laboratory, 26221 Patras, Greece; 3 Mastorakou Private Laboratory, 26221 Patras, Greece; 4 Adamopoulou Private Clinic, 26222 Patras, Greece; 5 Kortesas Private Laboratory, 26334 Patras, Greece; 6 Dimitropoulou Private Laboratory, 26221 Patras, Greece; 7 Division of Pediatric Hematology-Oncology, First Department of Pediatrics, National and Kapodistrian University of Athens, 11527 Athens, Greece

**Keywords:** SARS-CoV-2, influenza A, vaccination, rapid tests, RT-PCR, young adults, carnival

## Abstract

Background: We investigated the impact of the indoor mass gathering of young people during the Patras Carnival in Greece on the course of the COVID-19 pandemic and the influenza A epidemic. Materials and Methods: For influenza A, we tested 331 subjects with high fever (>38 °C), who arrived at five separate private laboratories over a two-week period after the carnival, via rapid test. One hundred and eighty-eight of them were young adults (17–35 years old), all unvaccinated against influenza A but all immunized against SARS-CoV-2, either through vaccination or previous infection. For the SARS-CoV-2 pandemic, we tested 2062 subjects at two time periods, two weeks before and two weeks after the carnival, also via rapid test. Additionally, we examined 42 samples positive for influenza A and 51 samples positive for SARS-CoV-2 for the possibility of co-infection via molecular testing (i.e., RT-PCR). Results: 177/331 (53.5%) subjects tested positive for influenza A, and 109/177 (61.6%) of the positive subjects were young adults, and 93/109 (85.3%) of these subjects were tested in the first week after the carnival. Additionally, 42 samples of those subjects were molecularly tested, and 5 were found negative for influenza A but positive for SARS-CoV-2. Regarding the SARS-CoV-2 pandemic, the increase in the positivity index for young adults between the pre-carnival and post-carnival periods was moderate. Conclusions: Our study indicates that the indoor mass gathering of young people during the carnival contributed to the outbreak of an influenza A epidemic and had a moderate but not statistically significant impact on the course of the SARS-CoV-2 pandemic, corroborating the crucial role of vaccination against the epidemic’s waves. It also showed the need for the use of high-quality rapid tests for their management.

## 1. Introduction

The SARS-CoV-2 virus was first recorded in the city of Wuhan in December 2019 and spread rapidly throughout the globe, leading the WHO to declare a global pandemic on 11 March 2020 [1]. The first confirmed case in Greece was recorded on 26 February 2020, leading to the first set of restrictive measures, including a nationwide lockdown on 23 March 2020, a large-scale public awareness campaign, and widespread mandatory mask use, being implemented [2].

Seasonal influenza A normally occurs in Europe in the form of an epidemic in the period from November to April each year [3]. The COVID-19 pandemic and the subsequently unprecedented widespread restrictive measures that were implemented resulted in the loss of the epidemic character of influenza A, with its presence in the community being limited to sporadic cases [4].

On the other hand, the major shift in focus by policy and health authorities toward a SARS-CoV-2 vaccination strategy resulted in the inadequate awareness of the importance of influenza A vaccination, leaving a significant proportion of the general population, mainly young people, unvaccinated against influenza A [5,6].

The carnival in Greece took place between the 5th and 7th of March 2022. The carnival celebration in the city of Patras is considered the most popular nationwide, and it is an attraction for many people from different parts of the country (e.g., hotels reported a 90% occupancy rate). This was the first occasion after two years of the pandemic that the Greek government allowed a major cultural event to take place without any restrictive measures. This resulted in the mass gathering of young people, the vast majority 17–35 years old, in indoor entertainment venues—mainly nightclubs—without any measures of personal protection.

In the two weeks that followed the carnival, a significant increase in recorded cases of influenza A was observed both in Patras and nationwide as well as an increase in cases of COVID-19, as reported by the Greek National Public Health Organization (NPHO) [7,8].

The aim of the present study was to assess the epidemiological effects of the carnival period and the mass gathering of young adults that occurred on the epidemic of influenza A and the course of the COVID-19 pandemic through the collection of data in outpatient laboratories, where most of the asymptomatic patients or patients not presenting with severe symptoms tended to get tested.

## 2. Material and Methods

### 2.1. Participating Laboratories

The data for the study were collected from five private diagnostic laboratories and covered a two-week period before (20 February 2022–5 March 2022) and a two-week period after the Patras Carnival (8 March 2022–20 March 2022). All laboratories were located in the center of the city of Patras and met all necessary criteria for carrying out rapid tests and RT-PCR analyses for influenza A and SARS-CoV-2.

### 2.2. Subject Enrollment

#### 2.2.1. Influenza A Subjects

For the study of influenza A, 331 subjects, who came to the abovementioned laboratories during the first two weeks after the carnival (8 March 2022–20 March 2022) were tested. The main reported symptom was high fever (>38 °C), with accompanying symptoms of headache and pharyngalgia. The criteria for their enrollment were a preexisting negative (−) self-test and/or rapid test for COVID-19 and instructions by their doctor to be tested for influenza A. 

As we aimed to investigate the impact of the carnival, especially the crowding of young adults inside nightclubs, on the outbreak of influenza A, the subjects were divided into groups A and B: group A (188 people) included 17–35 year old subjects who were considered (based on social and cultural factors) people that almost exclusively visited nightclubs during the carnival period. Group B included subjects >35 and <17 years old (143 subjects) who were considered people that were the least likely to have visited nightclubs during the carnival and were used as a control group (Table 1 and Table 2). Comparison of the positivity indices of patients >35 years old and patients <17 years old showed that they did not differ significantly at any given timepoint. One hundred and twenty-three subjects were male and two hundred and eight were female. 

An additional nasopharyngeal smear was obtained and placed in a 3 mL VTM transport medium to be further tested through RT-PCR for 42 subjects in group A who were found positive for influenza.

Clinical information was obtained from the subjects in group A, who were all immunized against SARS-CoV-2 (via vaccination or previous infection), but none were vaccinated against influenza A.

#### 2.2.2. SARS-CoV-2 Subjects

For the study of the COVID-19 pandemic, 2065 subjects were tested in total, of which 980 subjects were male and 1082 were female. The main reason for the visit to our laboratories was a government-mandated SARS-CoV-2 rapid test for all unvaccinated people, necessary to go to work or to visit public services. Another reason was to investigate the possibility of a SARS-CoV-2 infection because of close contact with a confirmed case and/or the appearance of mild symptoms (such as low-grade fever, headache, and pharyngalgia). It should be noted that none of these subjects were tested for influenza A. 

To study the impact of the mass gathering on the course of the COVID-19 pandemic, these subjects were divided into groups based on age and time of arrival and taking the following facts into account:

- The national positivity index for SARS-CoV-2 was stable for all ages in the last 2 months before the carnival;
- The last two weeks before the carnival, there was no known event in our town responsible for a mass gathering;
- The mass gathering during the carnival involved almost exclusively young adults 17–35 years old;
- The Omicron variant has an incubation period of approximately 3 days [9,10], while the carnival lasted for 3 days (5 March 2022–7 March 2022).

Considering the above facts as well as the distinction criteria used in the influenza A study, the subjects were divided into 4 subgroups. Groups C1 and C2 included subjects who came to our laboratories during the two-week pre-carnival period. Group C1 included 17–35 year old young adults (319 subjects), and group C2 included subjects >35 and <17 years old as a non-young adult control group (617 subjects). Groups D1 and D2 included subjects who came to our laboratories during the two-week post-carnival period. Group D1 included 17–35 year old young adults (414 subjects), and group D2 included subjects >35 and <17 years old as a non-young adult control group (715 subjects) (Table 1 and Table 2). 

An additional nasopharyngeal smear was obtained and placed in a 3 mL VTM transport medium to be further tested by the RT-PCR method from 51 subjects in groups D1 and D2 who tested positive for SARS-CoV-2.

No further clinical information (e.g., immunization status) was obtained from these subjects.

### 2.3. Procedures

For the diagnosis of influenza A, the kit “Influenza A&B Ag Rapid Test” (Healgen Scientific, Houston, TX, USA) rapid test was used, which simultaneously controls for the presence of influenza A and B capsular antigens. For the diagnosis of COVID-19, the kit “Rapid SARS-CoV-2 Antigen Test Card” (Xiamen Boson Biotech, Xiamen, China) was used, which controls for the presence of the SARS-CoV-2 capsular antigen. A nasopharyngeal smear was obtained from both nostrils, and the samples were processed according to the kits’ instructions.

Additionally, 42 group A subjects positive for influenza A as well as 51 subjects in groups D1 and D2 positive for SARS-CoV-2 were molecularly tested through RT-PCR for the detection of the H1N1 and H3N2 subtypes of influenza A and SARS-CoV-2. A nasopharyngeal smear was obtained and placed in a 3 mL VTM transport medium to be further tested through RT-PCR. The kits “Influenza virus A-type-FRT PCR kit” (Ecoli Dx, Praha, Czech Republic) and “ZENA SARS-CoV-2 Direct Detection Kit” (AMD, Nottingham, UK) were used according to their instructions, respectively.

### 2.4. Statistics

The R statistical software (version 4.2.1) was used for all statistical calculations. Odds ratios were used to estimate the relative risk of the subjects. Odds ratios are frequently used to represent the strength of the association between risk factors and binary outcomes. The odds ratio for a risk factor contributing to an outcome can be interpreted as whether a subject with the risk factor is more likely than someone without the risk factor to experience the outcome. Another feature of odds ratios is that it is easy to test the statistical strength of the association. Odds ratios are typically reported in a table with 95% CIs. If the 95% CI for an odds ratio does not include 1.0, then the odds ratio is statistically significant at the 5% level [11].

To investigate a possible relationship between age and time of onset of influenza A, the crude odds ratio (OR) [12] was used, dividing the odds of being infected with influenza A while belonging to group A (young adults) by the odds of being infected with influenza A while belonging to group B (non-young adults control group) during the first week, the second week, and overall. 

To investigate the impact of the mass gathering of young adults on the COVID-19 pandemic during the carnival, we used the crude odds ratio (OR), dividing the odds of a positive test for SARS-CoV-2 in the two weeks before the carnival by the odds of a positive test for SARS-CoV-2 in the two weeks after the carnival for each age group. In this way, we aimed to investigate whether the carnival had a significant impact on the SARS-CoV-2 positivity indices of the different age groups.

## 3. Results

### 3.1. Influenza A Rapid Tests

In the group of young adults 17–35 years old (group A), 109/188 subjects were positive for influenza A, while 3 subjects were positive for influenza B. This corresponds to a 57.9% positivity index for influenza A in this group. In the group of subjects <17 and >35 years of age (group B), 68/143 subjects tested positive for influenza A, which corresponds to a lower positivity index of 47.5%.

Additionally, based on the time of visit, 85.3% of subjects positive for influenza A in group A arrived during the first week of the outbreak (OR: 3.2), while 85.2% of subjects positive for influenza A in group B arrived during the second week. This corresponds to a significant OR of 3.2 for group A in the first week, which suggests that young adults had a major role in the onset of the influenza A outbreak (Table 3).

### 3.2. SARS-CoV-2 Rapid Tests

In the young adult group 17–35 years old, 51/319 were found positive in the pre-carnival period (group C1) and 81/414 in the post-carnival period (group D1), with a positivity index of 15.9% and 19.5%, respectively. This would be considered a moderate, albeit not statistically significant, increase in the positivity index as suggested by the OR of 1.28 (Table 4).

In subjects <17 and >35 years old, 71/617 subjects were found positive in the pre-carnival period (group C2) and 96/715 in the post-carnival period (group D2), with a positive index of 11.5% and 13.4% (*p*-value: 0.29), respectively. This was not a statistically significant increase, suggested as well by the lower OR of 1.19 (Table 4).

### 3.3. RT-PCR Tests

Forty-two samples in group A with a positive rapid test for influenza A were molecularly tested, and 37 samples were confirmed positive for influenza A, while 5 were negative for influenza A but positive for SARS-CoV-2. This corresponded to a false positive rate of 11.9%. Additionally, all samples truly positive for influenza A were negative for H1N1 and positive for H3Ν2.

Additionally, 51 samples from groups D1 and D2 with a positive rapid test for COVID-19 were molecularly tested and all were confirmed positive for SARS-CoV-2, while no simultaneous influenza A co-infection was detected in any of the samples.

## 4. Discussion

The Patras Carnival was the first time after two years of strict restrictions in Greece that a crowd of people gathered indoors without measures of personal protection. This seems to have led to the recurrence of influenza A with epidemic features, as evidenced by the high positivity index of the rapid tests performed in our laboratories, as well as the exacerbation of the SARS-CoV-2 pandemic.

In its initial phase, influenza A affected mainly young adults, since in the week of onset of the disease (8–13 March 2022), 85.3% of positive subjects belonged to the 17–35 year old age group, and there was a strong correlation between age and time of arrival as indicated by the OR (3.2) for this group. It should be noted that none of the laboratories recorded a suspected case of influenza A in the previous month before 8 March 2022 (8 February 2022–7 March 2022). Therefore, young adults could be considered as the “patient zero” of this influenza A epidemic in our city, and the focus of the spread were nightclubs, where many people were present without any measure of individual protection, with the lack of immunization coverage against influenza A in this group being a favorable factor. As the young adult subjects spread the infection among the rest of the community, the rate of infection increased in children <17 and populations >35 years old over the subsequent week.

In addition, molecular analysis in 42 samples showed that the strain of influenza A was of the H3N2 subtype, a fact that agrees with the official data of the National Public Health Organization (NPHO) and identifies the H3N2 subtype as being responsible for influenza A cases in Greece in the period 2021–2022 [8].

It is also important to note that molecular testing did not reveal any cases of coexisting influenza A and SARS-CoV-2 infection. Although cases of infection with both viruses have been reported [13,14,15], for the first time, to our knowledge, this has been investigated in outpatients, who were additionally vaccinated against SARS-CoV-2. The absence of co-infection may be due to the protection provided by the SARS-CoV-2 vaccine. On the other hand, it is worth noting the hypothesis of “viral interference” [16], where infection of respiratory cells by one virus prevents simultaneous infection by another. In fact, a study in animal models [17] showed that a previous influenza infection inhibits infection from coronaviruses, if the exposure occurs in a short time. Given that both viruses coexisted in the same environment (indoor entertainment venues) and at the same time, it is possible that influenza A infection acted as a deterrent to SARS-CoV-2 infection [18]. To further investigate this, a study with a larger number of outpatients—varying in age—in a time and place where infection of influenza A and SARS-CoV-2 coexisted is needed.

Regarding the COVID-19 pandemic, in the group of 17–35 year old subjects, a moderate increase in the number of new infections was observed between the pre-carnival and post-carnival periods (positivity index increase from 15.9% to 19.5% and an OR of 1.28). Meanwhile, a similar moderate increase was observed in the group of subjects <17 and >35 years old (positivity index from 11.5% to 13.4% and an OR of 1.19). This suggests that the impact of indoor mass gatherings during the carnival did not contribute significantly to the exacerbation of the pandemic. Considering the remarkable vaccination rate (75–80%) of young adults in our country [19], the aforementioned conclusion of our study is clear evidence that the mass vaccination of the general population is an effective method of containment that helped prevent a major outbreak of the COVID-19 pandemic. Additionally, as in the case of influenza A, the hypothesis of “viral interference” is something worth considering as a cause of preventing the outbreak of the COVID-19 pandemic (alone and/or synergistically with mass vaccination) but which needs to be explored with larger studies [20].

We also studied the performance of rapid tests for influenza A. We found that 5/42 samples that were found positive via rapid test were negative for influenza A but positive for SARS-CoV-2 through molecular testing, which is cause for concern. False positivity of rapid tests for influenza A has already been observed [21,22], although this is the first time, to our knowledge, that this kind of investigation has been conducted during a period when the two viruses coexisted with high prevalence. In our study, two different rapid test kits were used. In their enclosed instructions, in both cases, it was stated that they tested for specificity and cross-reactivity against the OC43 and/or the 229E coronavirus but not against SARS-CoV-2. This was also confirmed with three more rapid test kits used in our country (information regarding these kits are available upon request). This raises the serious question of the reliability of rapid tests for influenza A, which can lead to misdiagnosis during a period when COVID-19 and influenza A epidemics coexist (something we expect to happen during the next fall and winter).

## 5. Conclusions

The carnival provided the temporal and spatial conditions for studying the simultaneous public health interaction of two epidemic viruses: influenza A and SARS-CoV-2. Through our observations, the medical doctrine that the most effective way to stop any type of epidemic wave is to combine mass vaccination with individual protection measures and that only one of the two is, in most cases, not enough was further strengthened. It also seems that young adults tend to comply less with individual protective measures. On the other hand, the absence of cases of co-infection in subjects positive for influenza A, as well as the prevention of another major outbreak of the COVID-19 pandemic, can be counted as an additional benefit of vaccination against SARS-CoV-2, while at the same time bringing back to the fore the need for a systematic investigation of the hypothesis of “viral interference”. Finally, rapid tests proved to be extremely useful tools in the rapid and low-cost diagnostic management of two epidemics that occurred simultaneously but with the necessary condition that they meet high standards of specificity and accuracy.

## 6. Limitations of the Study

To our knowledge, this was a nonrandomized study. Therefore, its results exclusively regard the impact of mass gatherings on the course of influenza A and SARS-CoV-2 epidemics in our city’s population and especially in young adults. No other conclusions can be safely drawn concerning the status of these epidemics in the general population. In addition, the sample size that underwent molecular testing was small; therefore, the conclusions regarding the possibility of co-infection and the performance of rapid tests need to be confirmed with larger studies.

## Figures and Tables

**Table 1:** Subject enrollment.

Patient Group	Number of Subjects	Time Period	Type of Rapid Test
**Group A**	188	Two-Week Post-Carnival	Influenza A and B (Ag)
**Group B**	143	Two-Week Post-Carnival	Influenza A and B (Ag)
**Group C1**	319	Two-Week Pre-Carnival	SARS-CoV-2 (Ag)
**Group C2**	617	Two-Week Pre-Carnival	SARS-CoV-2 (Ag)
**Group D1**	414	Two-Week Post-Carnival	SARS-CoV-2 (Ag)
**Group D2**	715	Two-Week Post-Carnival	SARS-CoV-2 (Ag)

**Table 2:** Groups’ characteristics.

Patient Group	Age (Years)	Vaccination Status	Cause for Testing	Possibility of Attending Mass Gathering Events during the Carnival	Previous Testing
**Group A**	17–35	Only against SARS-CoV-2	Symptoms of Viral Infection ^a^	Very High	Negative (−) Rapid Test for SARS-CoV-2
**Group B**	<17 and >35	Only against SARS-CoV-2	Symptoms of Viral Infection ^a^	Very Low	Negative (−) rapid test for SARS-CoV-2
**Group C1**	17–35	Unknown	Variable ^b^	Very High	No
**Group C2**	<17 and >35	Unknown	Variable ^b^	Very Low	No
**Group D1**	17–35	Unknown	Variable ^b^	Very High	No
**Group D2**	<17 and >35	Unknown	Variable ^b^	Very Low	No

^a^ High fever (>38 °C), headache, and pharyngalgia. ^b^ Necessary to go to work or to visit public services, possibility of a SARS-CoV-2 infection because of close contact with a confirmed case, and/or the appearance of mild symptoms.

**Table 3:** Positivity indexes and odds ratios for subjects tested for influenza A.

	Positivity Index (%)	OR	95% CI	*p*-Value
**Group A**	57.9	1.87 1	1.21–2.90 -	*0.005*
**Group B (Reference Group)**	47.5			
**Group A—1st Week**	62.8	3.20 1	1.39–7.40 -	*0.006*
**Group B—1st Week (Reference Group)**	34.5			
**Group A—2nd Week**	42.1	1.00 1	0.45–2.18 -	1.00
**Group B—2nd Week (Reference Group)**	50.0			

**Table 4:** Positivity indexes and odds ratios for subjects tested for SARS-CoV-2.

	Positivity Index (%)	OR	95% CI	*p*-Value
**Group C1 (Reference Group)**	15.9	1 1.28	- 0.86–1.88	0.21
**Group D1**	19.5			
**Group C2 (Reference Group)**	11.5	1 1.19	- 0.86–1.65	0.29
**Group D2**	13.4			
